# Loss of *BRCA1* promotor hypermethylation in recurrent high-grade ovarian cancer

**DOI:** 10.18632/oncotarget.20945

**Published:** 2017-09-15

**Authors:** Katharina Prieske, Stefan Prieske, Simon A. Joosse, Fabian Trillsch, Donata Grimm, Eike Burandt, Sven Mahner, Barbara Schmalfeldt, Karin Milde-Langosch, Leticia Oliveira-Ferrer, Linn Woelber

**Affiliations:** ^1^ Department of Gynecology and Gynecologic Oncology, University Medical Centre Hamburg-Eppendorf, 20246 Hamburg, Germany; ^2^ Department of Tumor Biology, University Medical Centre Hamburg-Eppendorf, 20246 Hamburg, Germany; ^3^ Department of Gynecology and Obstetrics, University of Munich, 81377 Munich, Germany; ^4^ Department of Pathology, University Medical Centre Hamburg-Eppendorf, 20246 Hamburg, Germany

**Keywords:** BRCA methylation, ovarian cancer, platinum, recurrence, high-grade

## Abstract

**Background:**

Approximately 20-25% of ovarian cancers are attributable to germline or somatic *BRCA1/2* mutations, resulting in defects in the homologous recombination pathway. Inactivation of these genes can also be mediated by epigenetic changes, e.g., hypermethylation of CpG islands in the promoter regions. In such homologous recombination deficient tumors, platinum based chemotherapy is in general effective, however, loss of hypermethylation might lead to refractory disease. The aim of this study was to evaluate the stability of *BRCA1* promoter hypermethylation in recurrent disease after platinum based chemotherapy.

**Methods:**

Tumor tissue from 76 patients with primary and 48 patients with platinum-sensitive recurrent high-grade ovarian cancer was collected. In a subgroup of 12 patients, ‘paired’ tumor tissue from primary and recurrent surgery was available. *BRCA1* promoter methylation status was assessed using methylation specific polymerase chain reaction and was verified by Sanger Sequencing.

**Results:**

73.7% (56/76) of primary and 20.8% (10/48) of recurrent tumors displayed *BRCA1* promoter hypermethylation. *BRCA1* promoter methylation status was not associated with progression-free- or overall survival. In the paired subgroup 83.3% (10/12) of the primary vs. 16.7% (2/12) of the recurrent tumors showed hypermethylation. In eight patients loss of *BRCA1* hypermethylation was observed, whereas two patients had stable methylation status.

**Conclusions:**

Loss of *BRCA1* promoter methylation may be a mechanism to restore *BRCA1* function in recurrent disease. However, currently the clinical significance is still unclear and should be evaluated in prospective clinical trials.

## INTRODUCTION

Approximately 15-20% of ovarian cancers are attributable to germline *BRCA1/2* mutations resulting in defects in the homologous recombination (HR) pathway [1, 2]. These tumors share a distinct phenotype with high response rates to platinum based chemotherapy, long disease free intervals and improved overall survival (OS). In an analysis by ‘the cancer genome project’ (TCGA) [1] it has been reported that beyond *BRCA1/2* germline mutations an estimated 30-35% of high-grade serous ovarian carcinomas harbour molecular defects in the HR pathway, including somatic mutations and epigenetic alterations. This has been termed “*BRCAness*” as these tumors share phenotypic characteristics with hereditary determined ovarian carcinomas. Somatic mutations have been found in numerous HR associated genes (e.g., *BRCA1, BRCA2, ATM, BARD1, BRIP1, CHEK1, CHEK2, FAM175A, MRE11A, NBN, PALB2, RAD51C,* and *RAD51D*) [2] being highly predictive of primary platinum sensitivity and improved OS. Besides germline and somatic mutations, epigenetic modifications like promoter hypermethylation of *BRCA1* can also lead to downregulation of gene expression [3, 4] and thus silencing of the *BRCA1*. Methylation of CpG islands in the *BRCA1* promoter of primary high-grade ovarian carcinomas has been described ranging from 11-89% [1, 5-8]. The TCGA reported that epigenetic silencing of *BRCA1* is mutually exclusive of *BRCA1/2* mutations [1]. As results regarding the impact of *BRCA1* silencing by promoter hypermethylation on progression free survival (PFS) or OS vary [1, 5, 9], the stability and clinical significance of this alteration remains currently unclear. The aim of this study was therefore to investigate the stability of *BRCA1* promoter hypermethylation in platinum sensitive recurrent disease after platinum based chemotherapy.

## RESULTS

### *BRCA1* promoter hypermethylation analysis on primary and recurrence tumors

**Figure 1:**
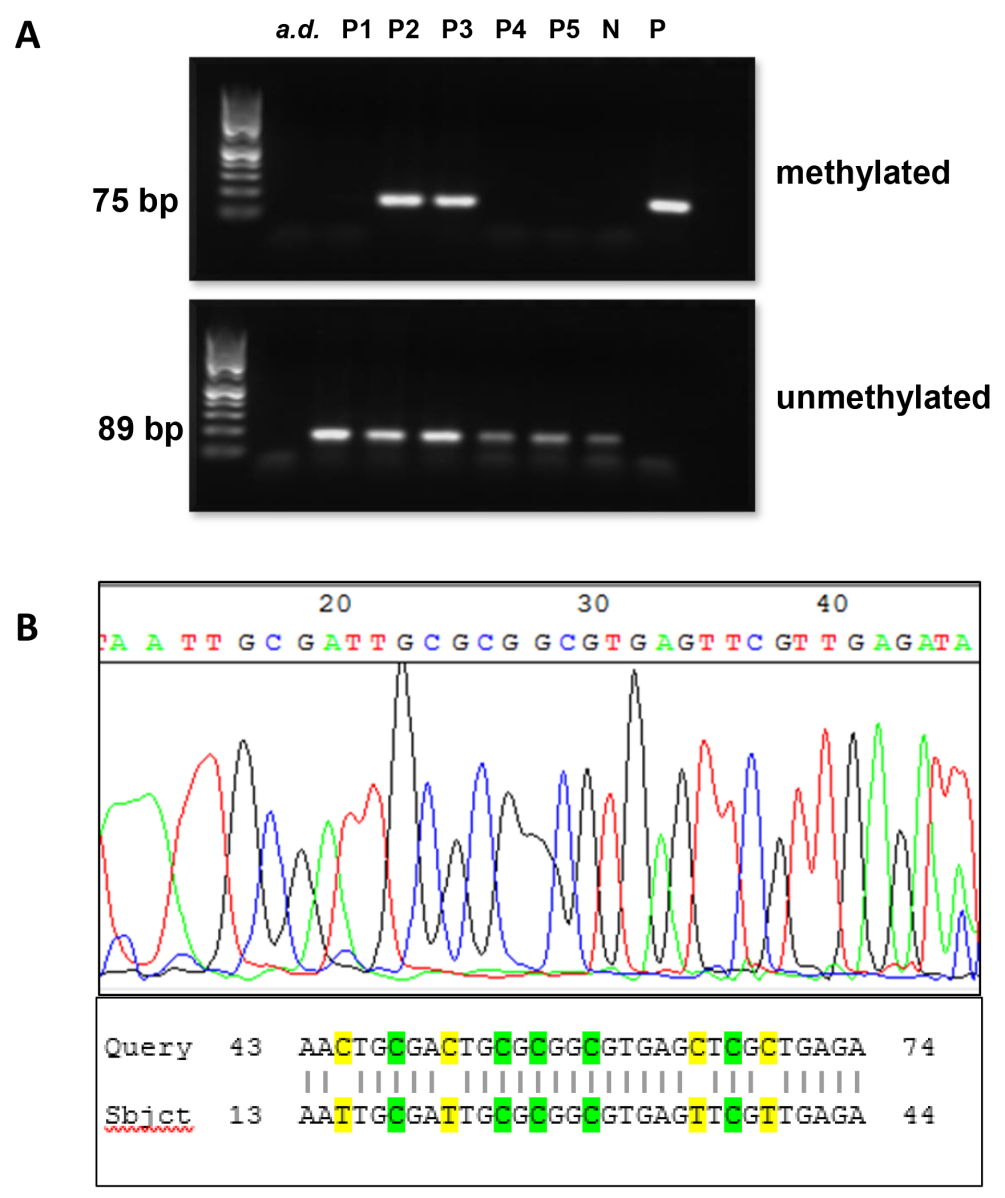
Analysis of BRCA1 promoter methylation status by MS-PCR and sanger sequencing **(A)** Methylation status of representative patient samples (P1-P5) determined by MS-PCR. Signals in the upper panel represent the presence of methylated DNA, whereas signals in the lower panel represent the presence of unmethylated DNA. MS-PCR controls: water (a.d.), genomic DNA from MDA-MB-231 cell line as negative control (N), universal methylated standard DNA as positive control (P). Patients 2 and 3 (P2/P3) showed *BRCA1* hypermethylation, whereas, *BRCA1* promotor in patients P1, P4 and P5 was unmethylated. Signals for unmethylated DNA were always seen as the tumor tissue samples always contained a small amount of normal cell. **(B)** Exemplary sequencing electropherogram of *BRCA1* reverse primer amplicon (upper panel). There are four hypermethylated CpG sites shown at position 18-19, 24-25, 26-27, 29-30, and 37-38 noticeable by the conservation of cytosine within the CpG site. The product of the Sanger sequencing compared to the primary sequence of the *BRCA1* promoter in BLAST (Basic Local Alignment Search Tool) (www.ncbi.nlm.nih.gov/BLAST) (lower panel). The conserved cytosines within the CpG sides are highlighted green at subject position 18, 24, 26, 29 and 37. The yellow marked bases at subject position 15, 21, 35 and 39 are former cytosines transformed into uracils and detected as thymine due to the bisulfite treatment.

*BRCA1* promoter hypermethylation was detected in 73.7% (56/76) of primary and 20.8% (10/48) of recurrent cases by MS-PCR analysis (p<0.0001). Detailed patients characteristics are presented in Table 1. DNA samples obtained from the MDA-MB-231 cell line were completely unmethylated at the *BRCA1* promoter. Examples of the MS-PCR analysis are shown in Figure 1A. To verify our results and quantify the number of methylated CpG sites, each sample positive for *BRCA1* hypermethylation in MS-PCR (n=66) was subjected to Sanger sequencing. A representative example is shown in Figure 1B. The MS-PCR product contained 9 CpG sites including the major transcription start site at 1581bp [10-12]. All hypermethylated cases (n=66) were highly methylated at all 9 CpG sites, confirming the results of MS-PCR. Nine randomly selected samples with unmethylated *BRCA1* promoter in MS-PCR were also analyzed by Sanger sequencing, none of them showed hypermethylation of the *BRCA1* promoter.

**Table 1:** Patient characteristics (primary group vs. recurrence group) yrs (years), FD (first diagnosis), RD (recurrent disease), LAE (lymphadenectomy), CTX (chemotherapy). The twelve pairs were included in the ‘primary’ and in the ‘recurrent’ group

Characteristics	Primary n=76	%	Recurrence n=48	%
**Age (yrs) at FD**				
Median (range)	63 (31-81)	n.a.	57 (31-71)	n.a.
**Age (yrs) at RD**				
Median (range)	65 (31-81)	n.a.	60 (31-78)	n.a.
**Lymph node status at FD**				
pN0	15	19.7	18	37.5
pN1	48	63.2	13	27.1
Nx (no LAE)	13	17.1	17	35.4
**FIGO stage at FD**				
IA	0	0	1	2.1
IIIA/IIIB	5	6.6	7	14.6
IIIC	47	61.8	33	68.7
IV	24	31.6	3	6.3
Unknown	0	0	4	8.3
**Grading**				
High grade	76	100	48	100
**Distant metastasis at FD**				
M0	52	68.4	40	83.3
M1	24	31.6	2	4.2
Pleura	7	9.2	1	2.1
Liver	10	13.2	1	2.1
Lung	2	2.6	0	0
Unknown	5	6.6	0	0
Unknown	0	0	6	12.5
**Histology**				
Serous	62	81.6	43	89.6
Others	14	18.4	5	10.4
Undifferentiated	6	7.9	0	0
Clear cell	4	5.3	1	2.1
Mixed	2	2.6	1	2.1
Endometrioid	1	1.3	1	2.1
Mucinous	1	1.3	2	4.2
**Neoadjuvant CTX**				
No	63	82.9	40	83.3
Yes	13	17.1	5	10.4
Unknown	0	0	3	6.3
**Residual tumor at FD**				
Microscopic	41	53.9	35	72.9
<1cm	11	14.6	3	6.3
>1cm	22	28.9	3	6.3
unknown	2	2.6	7	14.6
**Adjuvant CTX at FD**				
Platinum based combinations	70	92.1	41	85.4
Carboplatin only	4	5.3	1	2.1
No chemotherapy	2	2.6	5	10.4
Unknown	0	0	1	2.1
**Recurrence status**				
First recurrence	76	100	48	100
**Time to recurrence (months)**				
Median (range)	16.5 (2-67)	n.a.	23 (7-129)	n.a.
**Surgery at recurrence**				
Yes	26	34.2	48	100
Cytoreductive surgery	23	30.3	42	87.5
Palliative surgery	2	2.6	4	8.3
Intention unknown	1	1.3	2	4.2
Not performed	50	65.8	0	0
**Follow up from FD (months)**				
Median (range)	39 (2-124)	n.a.	55.5 (7-156)	n.a.

Besides age at diagnosis, none of the clinicopathological variables tested in univariate analysis (FIGO stage, residual tumor, lymph node status, neoadjuvant chemotherapy and distant metastasis) correlated with *BRCA1* promoter hypermethylation (Table 2). Results did not change if stratified for primary and recurrent tumors (data not shown). Dividing the total primary cohort into the different histological subtypes, 44 (70.9%) of the high-grade serous tumors showed *BRCA1* promoter hypermethylation, as well as four undifferentiated (66.6%), four clear cell (100%), two mixed (100%), one endometrioid (100%) and one mucinous (100%) tumor sample.

**Table 2:** Correlation between methylation status and clinical parameters for the total group yrs (years), FD (first diagnosis), RD (recurrent disease), CTX (chemotherapy)

Characteristics total cohort n=124	Methylated n=66	%	Unmethylated n=58	%	*p* value
**Age (yrs) at FD**					
Median (range)	63 (34-81)	n.a.	58 (31-75)	n.a.	0.029
**Age (yrs) at RD**					
Median (range)	65 (38-81)	n.a.	61 (31-77)	n.a.	
**Lymph node status at FD**					
pN0	18	27.3	15	25.9	*0.525*
pN1	38	57.6	24	41.4	
Nx	10	15.2	19	32.6	
**FIGO stage at FD**					
IA	0	0	1	1.7	*0.476*
IIIA/IIIB	8	12.1	4	6.9	
IIIC	41	62.1	39	67.2	
IV	16	24.2	11	19.0	
unknown	1	1.5	3	5.2	
**Grading**					
High grade	66	100	58	100	*0.584*
**Distant metastasis**					
M0	48	72.7	44	75.9	*0.397*
M1	16	24.2	10	17.2	
unknown	2	3.0	4	6.9	
**Histology**					
Serous	54	81.8	51	88	*0.733*
Others	12	18.2	7	12.1	
Undifferentiated	4		2		
Clear cell	4		1		
Mixed	2		1		
Endometrioid	1		1		
Mucinous	1		2		
**Neoadjuvant CTX at FD**					
No	54	81.9	49	84.5	*0.263*
yes	12	18.2	6	10.3	
unknown	0	0	3	5.2	
**Residual tumour (FD)**					
microscopic	38	57.6	38	65.5	*0.279*
<1cm	17	25.8	8	13.8	
>1cm	7	10.6	7	12.1	
unknown	4	6.1	5	8.6	

**Figure 2:**
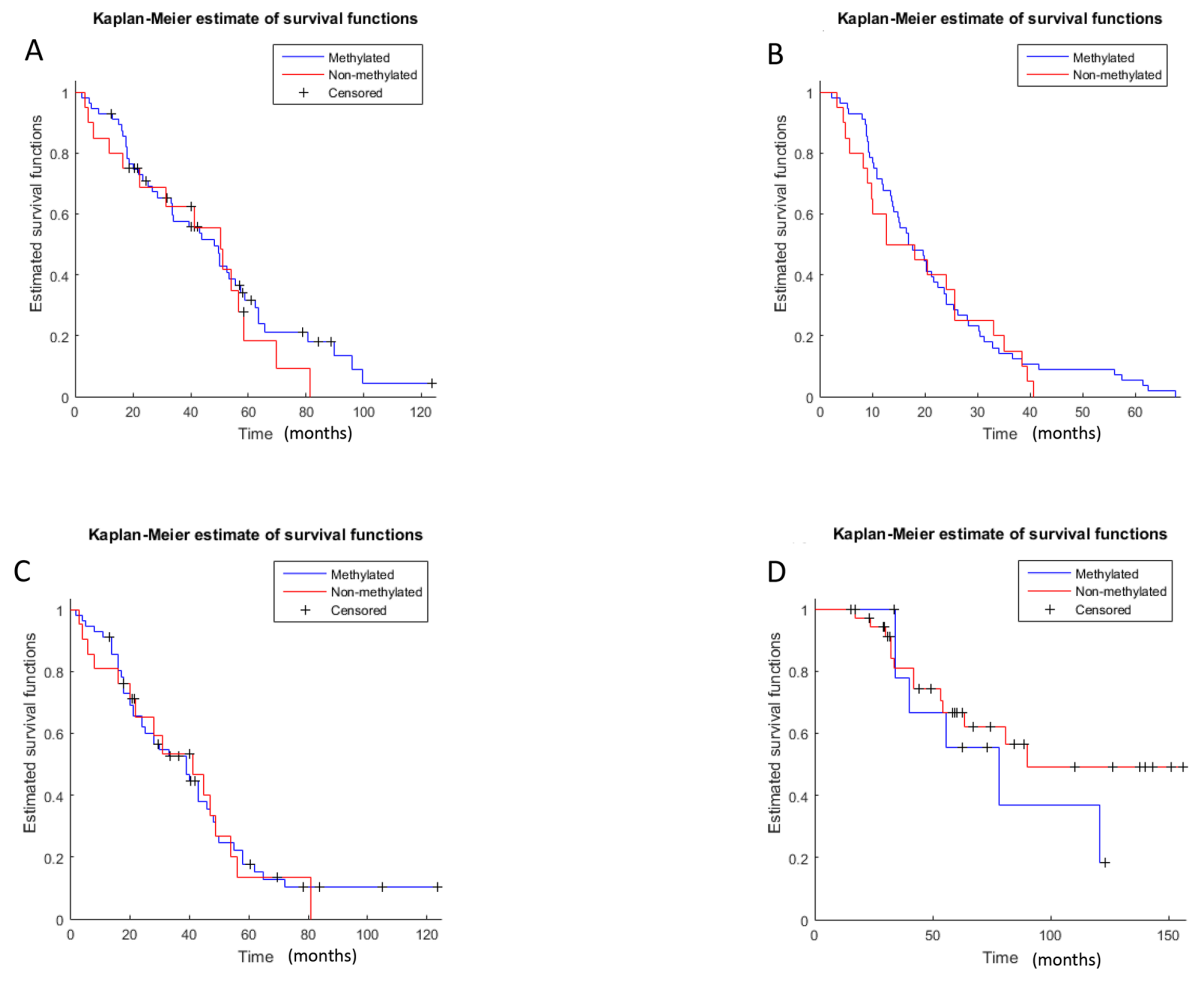

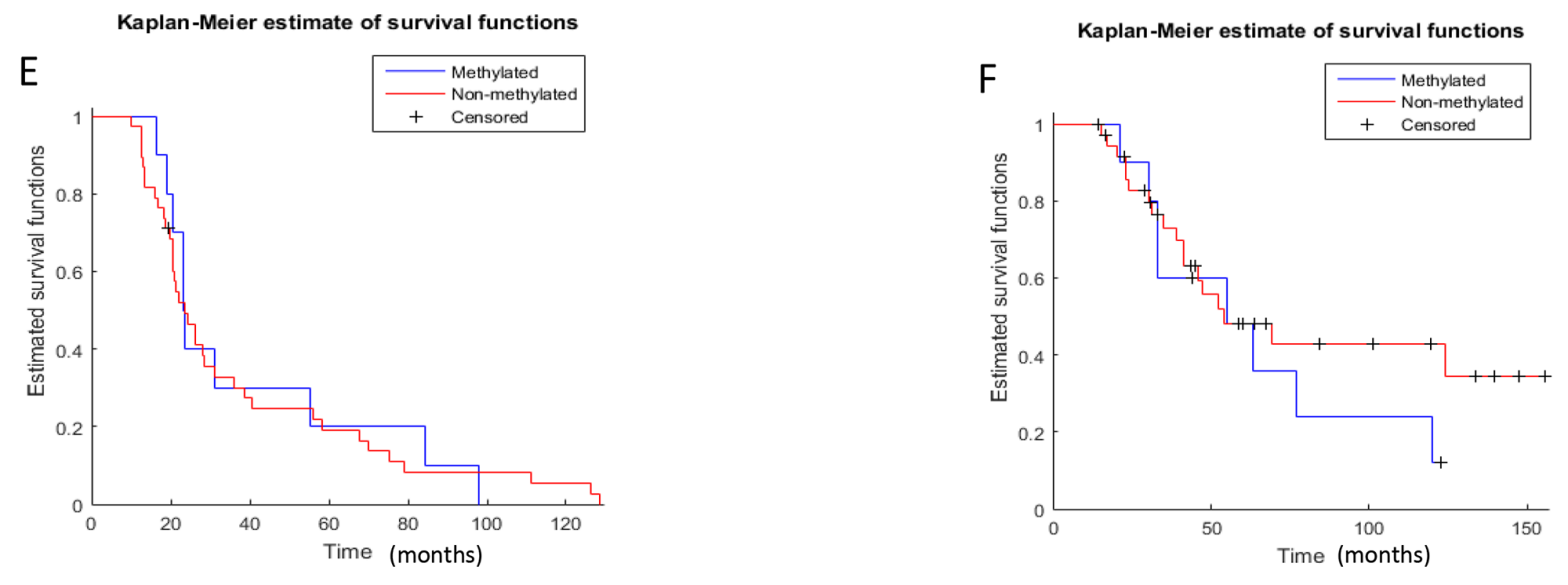
Kaplan-Meier survival estimates **(A)**: OS primary group: methlylated vs. non-methylated (p=0.239); **(B)**: PFS primary group: methlylated vs. non-methylated (p=0.305); **(C)**: PFS2 primary group: methlylated vs. non-methylated (p=0.43); **(D)**: OS recurrence group: methlylated vs. non-methylated, (p=0.283); **(E)**: PFS recurrence group: methlylated vs. non-methylated (p=0.485); **(F)** PFS2 primary group: methlylated vs. non-methylated (p=0.22); OS: overall survival, PFS: progression free survival; PFS2: progression free survival 2 (time to next treatment).

Hypermethylation of the *BRCA1* promoter did not correlate with OS or PFS in both groups (OS primary: p=0.24, methylated 46.75 months vs. unmethylated 48.6 months; HR: 0.79, 95%CI: 0.43-1.43; OS recurrent: p=0.28, methylated 71.3 months vs. unmethylated 89.6 months, HR: 1.5, 95%CI:0.56-3.94; PFS primary: p=0.30, methylated 16.8 months vs. unmethylated 12.7 months, HR: 0.86, 95%CI: 0.51-1.44, PFS recurrent: p=0.48, methylated 23.1 months vs. unmethylated 22.8 months, HR: 0.95, 95%CI: 0.47-1.92; Figure 2, Table 3). Overall, there was no statistically significant difference in PFS and OS between the methylated and unmethylated tumors corrected for tumor stage (OS: p=0.76; HR: 0.93 95% CI: 0.59-1.46; PFS: p=0.35, HR: 1.29, 95%CI:0.75-2.22; Cox proportional hazards regression). Time to next progression (PFS2) was evaluated for both groups (primary and recurrent cases), as well. No statistically significant difference in PFS2 was detected (PFS2 primary: p=0.43, methylated 36.2 months vs. unmethylated 40 months, HR: 0.93, 95% CI: 0.52-1.6; PFS2 recurrent: p=0.22, methylated 51.3 months vs. unmethylated 53.1 months, HR: 1.51, 95% CI: 0.52-1.6), Table 3.

**Table 3:** PFS, PFS2 and OS in primary and recurrent methylated vs. unmethylated ovarian cancer OS (overall survival), PFS (progression-free survival), PFS2 (progression free survival 2, time to next treatment) primary (primary tumor group), recurrent (recurrent tumor group), methylated (*BRACA1* promoter methylation), unmehtylated (no *BRACA1* promoter methylation), pts.: patients

Primary	Methylated	Unmethylated	
PFS (months), pts.	16.8, n=56	15.3, n=21	p=0.29, HR: 0.85, (95%CI: 0.51-1.4)
PFS2 (months), pts.	36.2, n=45	40, n=16	p=0.43, HR: 0.93 (95%CI: 0.52-1.6)
OS (months), pts.	46.75, n=56	50.6, n=21	p=0.24, HR:0.79, (95%CI: 0.44-1.42)

Recurrent	Methylated	Unmethylated	
PFS, pts. (months)	23.1, n=10	23.4, n=38	p=0.46, HR: 0.95, (95%CI: 0.48-1.97)
PFS2, pts. (months)	51.3, n=8	53.1, n=18	p=0.22, HR: 1.51, (95%CI: 0.52-1.6)
OS, pts. (months)	71.3, n=10	89.6, n=38	p=0.22, HR: 1.7, (95%CI: 0.63-4.48)

Thirteen patients in the primary group had received 3 cycles of neoadjuvant chemotherapy before tumor tissue was retained. The prevalence of *BRCA1* promoter methylation in this neoadjuvant treated group showed similar levels compared to the upfront debulking group [61.5% (8/13) vs. 76.2% (48/63); p=0.308, OR: 0.51 (95% CI 0.122-2.27)]. However, as no tissue was retained before treatment in the neoadjuvant group, no conclusion of potential changes of methylation after neoadjuvant chemotherapy can be drawn.

### Paired samples of primary and recurrent disease

In a subgroup of 12 patients, tumor tissue from primary and recurrent cytoreductive surgery of the same patient after first-line chemotherapy with carboplatin/paclitaxel was available. Patient characteristics are listed in Table 4.

**Table 4:** Patient characteristics ‘tumor pairs’ (n=12)yrs (years), first diagnosis (FD)

Characteristics	Pairs n=12	%
**Age (yrs) at FD**		
Median (range)	58.5 (30-69)	n.a.
**Lymph node status at FD**		
pN1	8	25
pN0	4	33.3
**FIGO stage at FD**		
IIIB	1	8.3
IIIC	9	75
IV	2	16.7
**Grading**		
High grade	12	100
**Histology**		
Serous	9	75
Endometrioid	1	8.3
Mixed	1	8.3
Clear cell	1	8.3
**Residual tumor at FD**		
Microscopic	9	75
<1cm	2	16.7
Unknown	1	8.3
**Surgery at recurrence**		
cytoreductive surgery	9	75
palliative surgery	3	25
**Time to recurrence (months)**		
median (range)	23 (9-67)	n.a.
**Follow up from FD (months)**		
median (range)	56.5 (16-89)	n.a.

**Figure 3:**
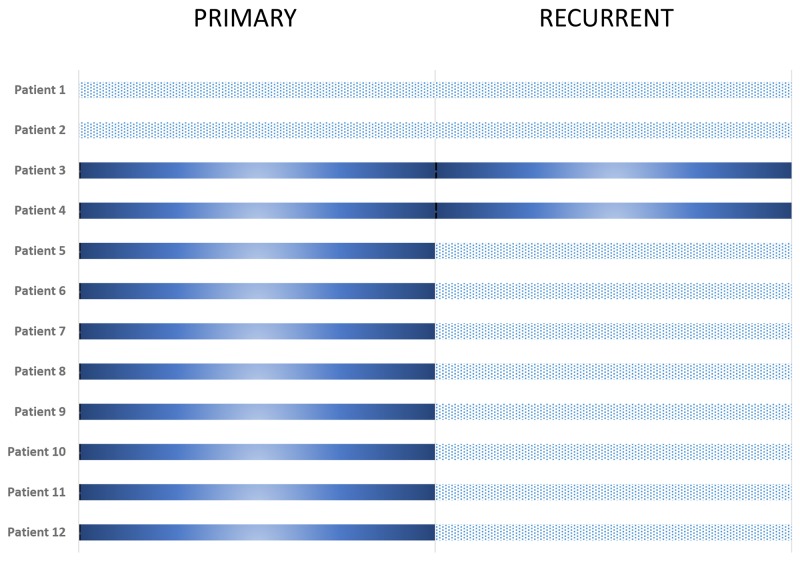
Methylation in ‘paired’ tumor samples Dark grey: BRCA1 promoter hypermethylated, light grey: BRCA1 promoter unmethylated.

*BRCA1* methylation level in this subgroup was similar as compared to the total cohort. 83.3% (n=10) of the primary carcinomas, vs. 16.7 % (n=2) of the paired recurrent tumors showed hypermethylation of the *BRCA1* promoter. Interestingly, in eight patients loss of *BRCA1* hypermethylation was observed, whereas two tumors had a stable *BRCA1* hypermethylation (Figure 3). None of the recurrent tumors showed a gain of *BRCA1* promoter hypermethylation. Of the methylated tumors seven were of serous histology, one endometrioid, one mixed and one clear cell.

## DISCUSSION

In the present study, a significantly lower rate of *BRCA1* promoter hypermethylation in recurrent ovarian cancer compared to primary tumors could be demonstrated. Most important, this change in *BRCA1* promoter hypermethylation could be confirmed in a subgroup of twelve primary and recurrent ovarian cancer tumor pairs after firstline platinum based chemotherapy in eight of ten patients.

*BRCA1* promoter hypermethylation has been described previously in primary high-grade serous ovarian cancer, methylation rates ranging from 10-89% [1, 5-8]. However, most studies report a methylation rate of 10-35% which is clearly below the 73% methylated cases in our cohort. Reasons for this discrepancy might be the relatively small patient cohorts and differences in methylation detection techniques. In our cohort, bias for a high rate of *BRCA1* promoter methylation might be the selection of exclusively high-grade histology as well as 100% relapsed ovarian carcinomas, also single centre selection bias cannot be ruled out. Furthermore, in our analysis most samples derived from FIGO IIIC/IV staged patients, whereas other groups had a higher percentage of FIGO stage I/II tumor samples [12]. Another difficulty in comparing *BRCA1* methylation levels, might have been that groups were looking at different CpG islands within the *BRCA1* promoter leading to variable *BRCA1* methylation rates.

In our study, primers were chosen as previously described, crossing the major transcription start site in the promoter of *BRCA1* at 1581 bp [11, 13]. Furthermore, in an analysis by Wilcox and colleagues who sequenced the proximal 660 bases of the *BRCA1* promoter [12], four CpG sites were found to be most frequently methylated. Two of these were covered by our primer set. These sites have been reported to be of particular interest, as their methylation strongly correlated with very low *BRCA1* expression [14]. The sites are located within and adjacent to a methylation sensitive v-Myb (**My**elo**b**lastosis) consensus binding site. With the elimination of transcription factor v-Myb binding, *BRCA1* expression is reduced [15]. Various groups have confirmed the correlation of *BRCA1* promoter methylation with reduction or loss of *BRCA1* mRNA- [3, 12] and protein [7, 9, 14] expression. CpG site 565 shows also high rates of methylation, however previous studies have shown, that this site is also highly methylated in normal cells [12, 16], so that we did not investigate this CpG island. MS-PCR is reported to be prone to false positive results due to nonspecific primer binding. Furthermore, it does not reveal the exact position and amount of CpG hypermethylation within a CpG island. Therefore, we analyzed the positive cases by Sanger Sequencing, which gives more detailed insight on the rate of hypermethylation, and could confirm all MS-PCR results by this method.

That *BRCA1* promoter hypermethylation does not predict response to platinum-based chemotherapy, even though it might contribute to tumor pathogenesis, is reflected in OS, PFS and PFS2 in our study, as no difference between the groups of patients with and without hypermethylation was observed. Accordingly, in a thorough analysis of 489 high-grade serous ovarian cancer samples, the TCGA did also fail to show an impact on OS for *BRCA1* methylated tumors [1]. These results have been confirmed by other groups as well [5, 9]. Taken together, these results suggest that hypermethylation is either heterogeneous or a dynamic process in ovarian cancer and may not be a good predictor for platinum-based chemotherapy. Methylation does not occur randomly, as methylation of CpG islands at particular genes can give the cancer cell a growth or survival advantage [17]. Many genes that are known to be methylated in cancers, can affect the hallmarks of cancer e.g. evasion of apoptosis, insensitivity to antigrowth signals, limitless replicative potential, sustained angiogenesis or DNA repair [18]. Not much is known about the selection of cancer cells under therapeutic pressure by tumor debulking and platinum-based chemotherapy with paclitaxel/carboplatin in ovarian cancer. However, it can be speculated that *BRCA1* promoter hypermethylation is heterogeneous within the tumor. Methylation of DNA repair genes during tumour development might lead to drug sensitivity, as disseminated single ovarian cancer tumor cells with functional *BRCA1* have a higher chemoresistance and might be selected for during therapy. Alternatively, as methylation in cytosine residues of CpG dinucleotides is an epigenetic alteration it seems plausible that demethylation of the *BRCA1* promoter occurs after interim chemotherapy and leads to reactivation of *BRCA1* mRNA expression and therefore to resistance of therapy.

Whereas tumors with *BRCA1*/*2* mutations (germline or somatic) show the most favourable outcome and response to the recently by the Food and Drug Administration (FDA) approved polyadenosine diphosphate [ADP]–ribose polymerase (PARP) inhibitors ‘olaparib’, ‘niraparib’ and ‘rucaparib’ [19-22] the clinical significance and response to *PARP* inhibitors in non *BRCA1/2* mutated tumors remains less clear. Tests to define ‘*BRCA*ness*’* and predict response to PARP inhibitors have been developed by different companies (e.g. Myriad MyChoice, Foundation Medicine) and applied in clinical trials. However, so far the applied tests were insufficient to identify patient cohorts who do not benefit from PARP inhibitors. Within the phase- III NOVA trial [19] Myriad MyChoice^®^ test was applied in to predict response to niraparib treatment, looking at whole-genome tumor loss of heterozygosity (LOH), telomeric allelic imbalance (TAI) and large-scale state transition (LST). However, in the NOVA trial a significant PFS benefit was observed in all predefined efficacy populations (*gBRCA1/2* mutation, non-gBRCA high HRD and non-gBRCA low HRD), especially also in the completely biomarker negative group. Therefore niraparib was approved by the FDA in 2016 for maintenance treatment of all ovarian cancer patients being in complete or partial remission to platinum based chemotherapy. The Foundation Medicine test was evaluated in the ARIEL II part I (Phase II) trial, detecting LOH only. PFS was significantly longer in the BRCA mutant and LOH high subgroups compared with the LOH low subgroup. Results from the ARIEL2 Part 1 trial indicate, that *BRCA1/2* wild-type tumors that have a high percentage of tumor genomic LOH, show an improved response to rucaparib treatment. Results from prospective validation in a phase III trial however, are still pending. Swisher and colleagues looked at BRCA1 methylation in archival and pre-treatment biopsies in the ARIEL 2 trial [23]. They observed a 31% decrease in BRCA1 methylation from archival to pre-treatment biopsies in recurrent disease (13 vs. 4 samples) after exposure to platinum chemotherapy. Most importantly, they could show that *BRCA1* methylation was associated with high LOH and sensitivity to rucaparib (duration of response: median 6.1 months for BRCA1 methylated cases, PFS 7.4 months for BRCA1 methylated cases, RECIST response: 52.4% 11/21 BRCA1 methylated cases). Accordingly, the authors concluded that if methylation was to be used as a predictor of PARP inhibitor sensitivity, it would need to be assessed in a pre-treatment specimen (not archival tissue).

Results from other groups that have evaluated methylation status over time have been obtained from very small groups. They all report on loss or stable *BRCA1* promoter methylation, whereas no study has reported on methylation gain in recurrent disease so far [7, 24, 25].

In conclusion, our study suggests that loss of *BRCA1* promoter methylation may be a mechanism to restore *BRCA1* function in recurrent disease. *BRCA1* promoter methylation might therefore not reflect in OS, PFS and PFS2 in these patients. However, clinical significance remains unclear and should be evaluated in prospective clinical trials.

## MATERIALS AND METHODS

### Patients and tumor tissue

Tumor tissue of 124 patients with high-grade ovarian cancer undergoing surgery at the University Medical Center Hamburg Eppendorf between 1993 and 2011 was analyzed, 76 primary (primary group) and 48 recurrent (recurrence group) cases were identified (Table 1). The tumor tissue was selected on the basis of histology and recurrence status. With the intention to minimise selection bias, only primary cases that had also suffered from recurrent disease were included in the analysis. All tissue samples in the recurrence group were from platinum sensitive ovarian cancer (recurrence free interval ≥ 6 months). From a subgroup of 12 patients ‘paired’ tumor tissue was available from the primary and recurrent cytoreductive surgery (Table 4). These twelve pairs were also included in the ‘primary’ and in the ‘recurrence’ group (Figure 1). Intermediate grade (G2) tumors were reviewed again by a gynecopathologist and were attributed low-or high-grade. All low-grade tumors were subsequently excluded. Nine tumors twere classified as G2 in original histology, but were then reevalutated and classified as high-grade by the gynecopathologist. Both two primary G2 tumors and 2/7 recurrent tumors showed *BRCA1* hypermethylation.

Tissue samples were obtained intraoperatively from within the abdominal cavity and were immediately snap frozen and stored at −80°C. Every sample was assessed on cryo-cut sections stained with haematoxylin and eosin. If necessary, stromal parts were removed to obtain at least 50% tumor cells in the sample used for DNA extraction. The majority of tissue samples had approximately 70% of tumor cells.

Informed consent was obtained from all included patients to access their tissue and review their clinical records according to our investigational review board and ethics committee guidelines (Ethics Committee of the Medical Board Hamburg reference number 190504). Data were retrieved from patient records and the institutional database providing information on clinicopathologic factors, histology and therapeutic approaches.

### DNA extraction

Tissue was disintegrated and genomic DNA was extracted using Precellys homogenizer (WVR International GmbH, Damstadt, Germany) and QIAamp DNA Mini Kit as well as All Prep Kit (Qiagen GmbH, Hilden Germany) according to the manufacturer’s instructions.

### Methylation-specific PCR (MS-PCR)

400ng of genomic DNA was modified by bisulfite conversion using EZ DNA Methylation Kit (Zymo Research Corp., Irvine, CA, USA) according to the manufacturer’s instructions. Methylation specific PCR was performed in a total volume of 25μl using the ZymoTaq DNA Polymerase (Zymo Research Corp., Irvine, CA, USA) and specific primers for either methylated or the modified unmethylated promotor region of the *BRCA1* gene, previously described by Esteller et al. [13]. Primer sequences for methylated *BRCA1* promoter DNA were 5’-TCG TGG TAA CGG AAA AGC GC-3’ and 5’-AAA TCT CAA CGA ACT CAC GCC G-3’ and primers for the unmethylated promoter were 5’-TTG GTT TTT GTG GTA ATG GAA AAG TGT-3’ and 5’-CAA AAA ATC TCA ACA AAC TCA CAC CA-3’. The lengths of the amplified products were 75bp (methylated) and 86bp (unmethylated). PCR conditions consist of an initial denaturing step of 10 minutes at 95°C followed by 35 cycles at 95°C for 30 seconds, 59°C for 30 seconds and 72°C for 1 minute, ending with a 7-minute final extension at 72°C. As positive control we used universal methylated DNA standard (Zymo Research Corp., Irvine, CA, USA) and as negative control DNA obtained from the MDA-MB-231 cell line, reported to be unmethylated in the *BRCA1* promoter [10]. The PCR products were separated on a 2% agarose gel.

### Sanger sequencing

MS-PCR results were confirmed by Sanger Sequencing using 400ng of genomic DNA, which was modified by bisulfite treatment and further amplified as described above. 20μl of the amplified product were used for sequencing (GATC Biotech, Konstanz, Germany) with the same primers used for PCR for forward as well as reverse reactions. The results were interpreted using the National Center for Biotechnology Information (NCBI) Basic Local Alignment Search Tool (BLAST) and compared to the methylation-specific PCR (Figure 1B).

### Statistical analysis

Association between clinical data [Fédération Internationale de Gynécologie et d’Obstétrique (FIGO), TNM] and methylation status, corrected for disease status (primary or recurrence), was assessed using binomial logistic regression. The survival and hazard functions were estimated by Kaplan-Meier estimator and the log-rank test, respectively. Analyses were performed in Matlab R2015a (The Mathworks). An alpha level of 0.05 was employed for rejecting the null-hypothesis.
